# Primitive neuroectodermal tumor of the kidney: case report and review of literature

**DOI:** 10.1186/1477-7819-10-279

**Published:** 2012-12-27

**Authors:** Chuanyu Sun, Zunguo Du, Shijun Tong, Ke Xu, Weihong Ding, Jianliang Sun, Qiang Ding

**Affiliations:** 1 Department of Urology, Huashan Hospital, FudanUniversity, Middle Wulumuqi Road 12, Shanghai, 200040, China; 2 Department of Pathology, Huashan Hospital, FudanUniversity, Shanghai, 200040, China

**Keywords:** Primitive neuroectodermal tumor, Kidney carcinoma, Ewing’s sarcoma

## Abstract

**Background:**

Renal primitive neuroectodermal tumor (rPNET) as a member of Ewing’s sarcoma family is extremely rare and usually occurs in children and young adults. Most literature about rPNET was isolated case reports.

**Case presentation:**

We reported a case of 45-year-old man with the complaint of right flank pain. Computerized tomography (CT) scan demonstrated a large substantive tumor involving the lower pole of the right kidney. Then the patient underwent radical nephrectomy. Pathologic characteristics and immunohistochemical analysis confirmed the diagnosis of rPNET. Additionally, the patient received three cycles of chemotherapy, and was still alive without metastasis at 15-months follow-up.

**Conclusion:**

rPNET is rare and presents aggressive clinical behavior and worse prognosis. We expect that further awareness and study of this rare tumor can be had by presenting our case.

## Background

Primitive neuroectodermal tumor (PNET) composed of small uniform round cells, is characterized by a translocation resulting in a fusion transcript of the EWS gene and ETS-related family of oncogenes [1]. PNET is presumed to be derived from neural crest, mostly presenting as bone or soft tissue masses in the trunk or axial skeleton in children and young adults [2]. Because of the morphologic overlap and the same genetic aberrations with Ewing’s sarcoma, PNET is now considered virtually the same entity as Ewing’s sarcoma [3]. Renal PNET (rPNET) as a member of Ewing’s sarcoma family is extremely rare [4]. Most literature about rPNET was isolated case reports. Recently, a case of 45-year-old man with rPNET was treated and followed up by us. Here we report this case and review the literature.

## Case presentation

**Figure 1:**
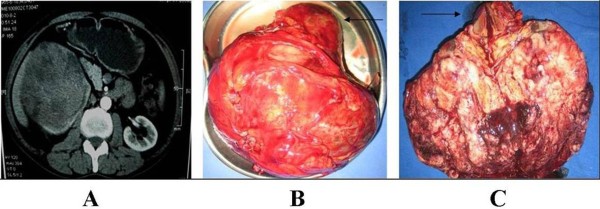
**The image and gross appearance of rPNET.** (**A**) CT scan of the kidney demonstrated a 12×10×10 cm substantive tumor involving the lower pole of the right kidney, then, after administration of the contrast medium, the tumor showed inhomogenous contrast enhancement with focal cystic and necrotic areas. (**B**) The substantive tumor measuring 13×13×9 cm replacing the lower pole of the right kidney was integratedly resected. (**C**) After slitting the specimen, a white sizable tumor with interspersed areas of hemorrhage and necrosis could be seen (arrow: the upper pole of the right kidney).

A 45-year-old man with the complaint of right flank pain for 1 week was admitted in August 2010. Ultrasonography showed a very large tumor on the right kidney. Computerized tomography (CT) scan showed a 12×10×10 cm substantive tumor involving the lower pole of the right kidney, while in the enhanced phase the tumor presented inhomogenous contrast enhancement with focal cystic and necrotic areas (Figure 1A). Urine examination showed occult blood (3+) and other laboratory examinations were normal. Chest X-ray and abdominal ultrasonography showed no evidence of tumor metastasis. Physical examination indicated a large and firm mass in the right abdomen.

The preoperative diagnosis of the patient was right renal carcinoma and radical nephrectomy was immediately done. The whole procedure was successful and the right kidney with the tumor was integratedly resected (Figure 1B). After slitting the specimen, a white sizable tumor measuring 13×13×9 cm with interspersed areas of hemorrhage and necrosis replacing the lower pole of the right kidney could be seen (Figure 1C).

**Figure 2:**
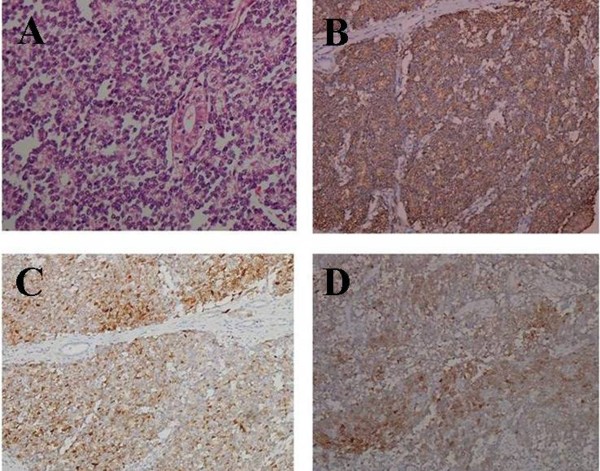
**HE and immunohistochemical staining of rPNET.** (**A**) The rosette-like structures formed by the small monotonous round cells. (**B**) The tumor cells showed strong positive expression of CD99. (**C**) The tumor cells showed positive expression of S-100. (**D**) The part of tumor cells showed positive expression of NSE.

The microscopic examination revealed that the tumor was composed of monotonous round cells with hyperchromatic round nucleus. The interspersed small dark cells indicating pyknosis of the tumor cells could form rosette-like structures (Figure 2A). The cytoplasm of the tumor cells was scanty, but, the rim of clear cytoplasm and discrete cell membranes were often apparent without extensive degenerative changes. Additionally, there were some small tumor emboli in the vessels of the kidney and the margin of ureter was negative. Most importantly, immunohistochemical staining indicated the positive expression of CD99, S-100, and neuron-specific enolase (NSE) in the tumor cells which supported the diagnosis of rPNET (Figure 2C, D, E).

The patient then underwent three cycles of chemotherapy (one cycle every 3 weeks). The chemotherapeutic agents included ifosfamide (2g, days 1 to 3) and epirubicin (100 mg, day 1). After the 15-months follow-up, the patient was still alive without metastasis.

## Discussion

rPNET first described by Mor *et al*. in 1975 is a very rare and aggressive malignant tumor [4]. rPNET usually occurs in children and young adults. Boys and men are more likely to suffer rPNET and the sex ratio (male:female) is about 3:1 [2]. The tumors tend to be very large and the maximum diameter of rPNET is always >10 cm [5-7]. So far, most literature about rPNET was isolated case reports and the largest case series including 79 patients with rPNET was described by Parham *et al*. in 2001 [8]. The age of these patients ranged from 2 months to 73 years old with a median age of 20 years [8].

The presenting symptoms and images of rPNET are non-specific and similar to other renal tumors. Therefore it is often difficult to distinguish rPNET from renal cell carcinoma and Wilm’s tumor [9]. However the images of rPNET are useful for staging of the disease. The diagnosis of rPNET mainly depends on pathologic characteristics and biomarkers. rPNET is characterized by small uniform round cells with dark nuclei, ill-defined cytoplasmic borders, and poorly-formed rosette-like structures [1,8]. Immunohistochemical staining of rPNET is always positive for different neural biomarkers such as S-100, Leu 7(HNK-1), and particularly NSE [4]. Additionally, CD99 also named MIC-2 antigen is crucial in the diagnosis of rPNET and the positive expression of CD99 has been demonstrated in more than 90% of rPNET [5]. But, CD99 is not specific and cannot be used as an absolute biomarker [10].

PENT is characterized by a translocation resulting in a fusion transcript of the EWS gene and ETS-related family of oncogenes [1]. Cytogenetic analyses may therefore be helpful in the diagnosis of rPENT. The translocation of t(11:22) (q24:q12) with the fusion transcript between the EWS gene (22q12) and the ETS-related oncogene (11q24) have been detected in more than 90% of the rPNET [5]. Several studies applied preoperative fine needle aspiration cytology to diagnose rPNET based on the constellation of cytomorphologic and immunohistochemical findings with subsequent confirmation by cytogenetic analyses [11-13].

rPNET appears to be an unique clinical entity that behaves more aggressively than PNET arising at other sites [6]. Approximately 20% to 50% of patients present with distant metastases, most commonly to regional lymph nodes, bone, bone marrow, lung, and liver [2]. The 5-year disease-free survival rate of rPNET is about 45% to 55% [4]. The overall survival in patients who had localized rPENT was longer than that in the patients who had rPENT with regional nodes or distant metastases [14]. The preferred treatment for rPENT is surgical resection associated with chemotherapy and radiotherapy treatment. The role of radiotherapy is not clear, but it may be advocated in locally advanced disease and involvement of Gerota’s fascia [14]. Postoperative chemotherapy for rPENT is usually used and can improve the prognosis of rPNET [6]. Most cases of rPNET may recur after nephrectomy without adjuvant chemotherapy. Severe multiple liver metastases occurred 6 months after radical nephrectomy in a 21-year-old man with rPENT who immediately underwent six cycles of chemotherapy with ifosfamide, etoposide, and adriamycin. After this treatment, residual tumor was removed and the tumor cells were absent histologically [6]. The most commonly used chemotherapeutics are adriamycin, etoposide, dactinomycin, vincristine, cyclophosphamide, and ifosfamide [4-7]. Several studies about combination therapy of surgery and chemotherapy for rPENT are summarized in Table 1.

**Table 1:** The combination therapy of surgery and chemotherapy for rPENT

**Patient**	**Age (years)**	**Gender**	**Diagnosis**	**Metastases**	**Treatment**	**Chemotherapeutic agents**	**Follow-up**	**Outcome**
1^4^	16	F	Right rPENT	No	Nephrectomy + chemotherapy	Ifosfamide Vincristine Vinblastine Dactinomycin Adriamycin	>4 years	Alive with complete remission
2^5^	26	F	Right rPENT	Lung metastasesat 2 monthsafter nephrectomy	Nephrectomy + chemotherapy, After metastases: sorafenib	Vincristine Adriamycin Cyclophosphamide Ifosfamide Etoposide	17 months	Alive with stabilized lung metastases
3^6^	21	M	Rihgt rPENT	Liver metastasesat 6 monthsafter nephrectomy	Nephrectomy,After metastases: Chemotherapy + partial hepatectomy	Doxorubicin Ifosfamide Etoposide	21 months	Alive
4^7^	9	M	Right rPENT	No	Nephrectomy + chemotherapy	Vincristine Doxorubicin Cyclophosphamide Ifosfamide Etoposide	10 months	Relapse in the paraspinal cervical region

This case presented with a relatively large localized tumor and had non-specific symptoms. Radical nephrectomy was immediately done based on the findings of CT of the right kidney. In this case, both the pathologic characteristic and the positive expression of CD99, S-100 and NSE in the tumor cells could support the diagnosis of rPNET. By reviewing the literature, the importance of combination therapy for rPENT was known. The patient underwent three cycles of chemotherapy including ifosfamide and epirubicin. After the 15-months follow-up, the patient was still alive without metastasis. Therefore, we suggest that postoperative adjuvant chemotherapy should be performed in cases of rPNET.

## Conclusion

rPNET is rare and presents with aggressive clinical behavior and worse prognosis. Immunohistochemical staining for CD99 and some neural biomarkers along with cytogenetic studies play a great role in the diagnosis of rPNET. Radical nephrectomy combined with chemotherapy and radiotherapy is the recommended treatment for rPENT. We expect that further awareness of this rare tumor can be had by presenting our case.

## Consent

Written informed consent was obtained from the patient for publication of this case report and any accompanying images. A copy of the written consent is available for review by the Series Editor of this journal.

## Abbreviations

CT: Computerized tomography; NSE: Neuron specific enolase; PNET: Primitive neuroectodermal tumor; rPNET: Renal primitive neuroectodermal tumor.

## Competing interests

The authors declare that they have no competing interests.

## Authors’ contributions

CS drafted the manuscript. SJ and JL did the operation for the patient. KX and WD collected the materials. ZD provided the pathological figures. All authors read and approved the final manuscript.
